# Supplementary material for: Lipidomic biomarkers in plasma correlate with disease severity in adrenoleukodystrophy
Source: Commun Med (Lond). 2024 Sep 10;4:175. doi: 10.1038/s43856-024-00605-9 (PMC11387402; doi:10.1038/s43856-024-00605-9)
Supplement: Supplementary file 2 — Supplementary Figs. [file 43856_2024_605_MOESM2_ESM.pdf]

Supplemental Figure 1

Figure 1A: Male ALD patients without cerebral ALD

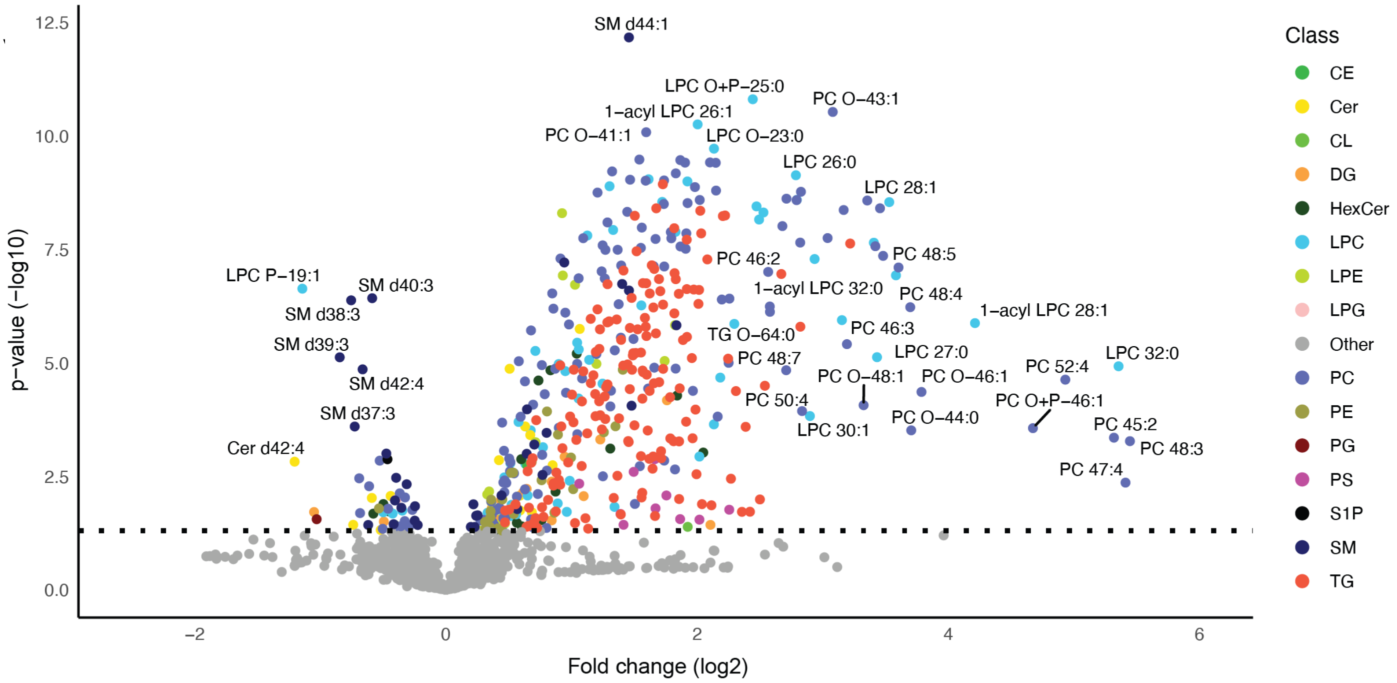

Legend: Volcano plot of lipid levels in male ALD patients without cerebral ALD versus controls. The vertical axis contains the p-value (−log10), and the horizontal axis the fold change (log2). Colored dots are lipids with a p-value of <0.05.

Figure 1B: Male ALD patients without adrenal insufficiency versus controls

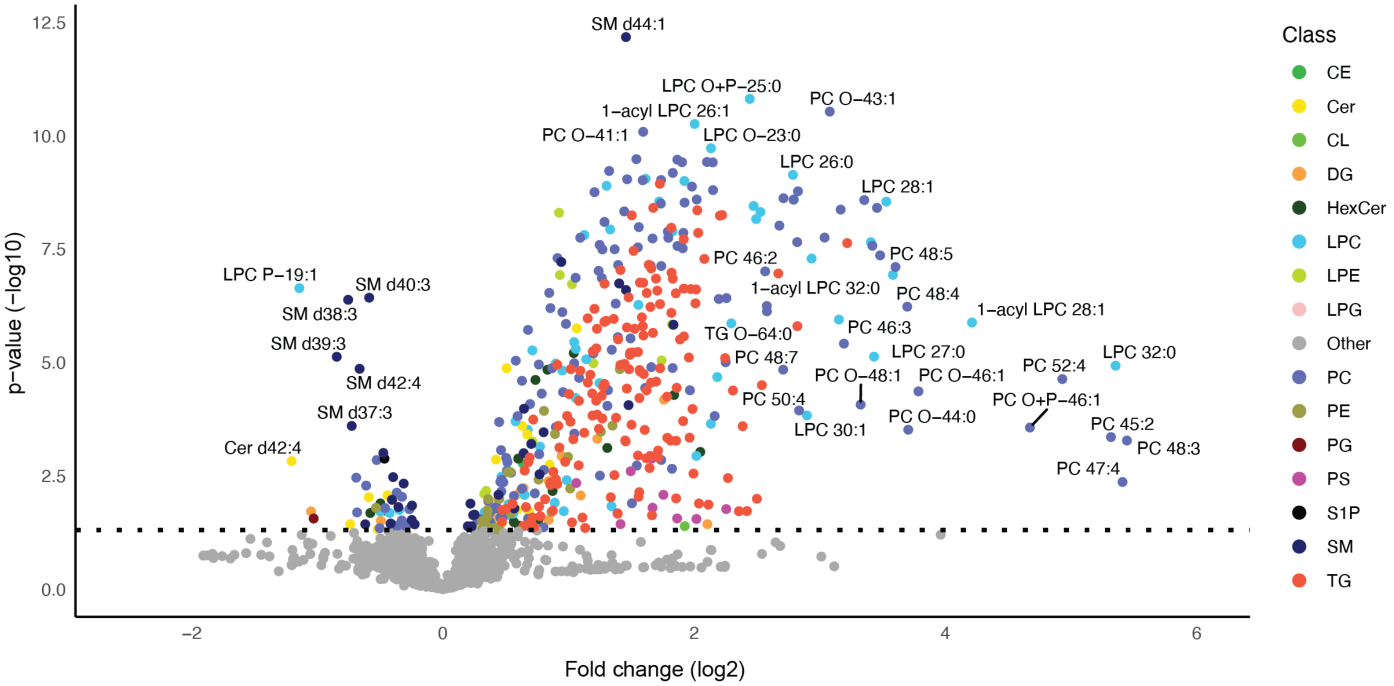

Legend: Volcano plot of lipid levels in male ALD patients without adrenal insufficiency versus controls. The vertical axis contains the p-value (−log10), and the horizontal axis the fold change (log2). Colored dots are lipids with a p-value of <0.05.

Figure 1C: Male ALD patients with mild spinal cord disease (EDSS≤6) and aged >55 years versus

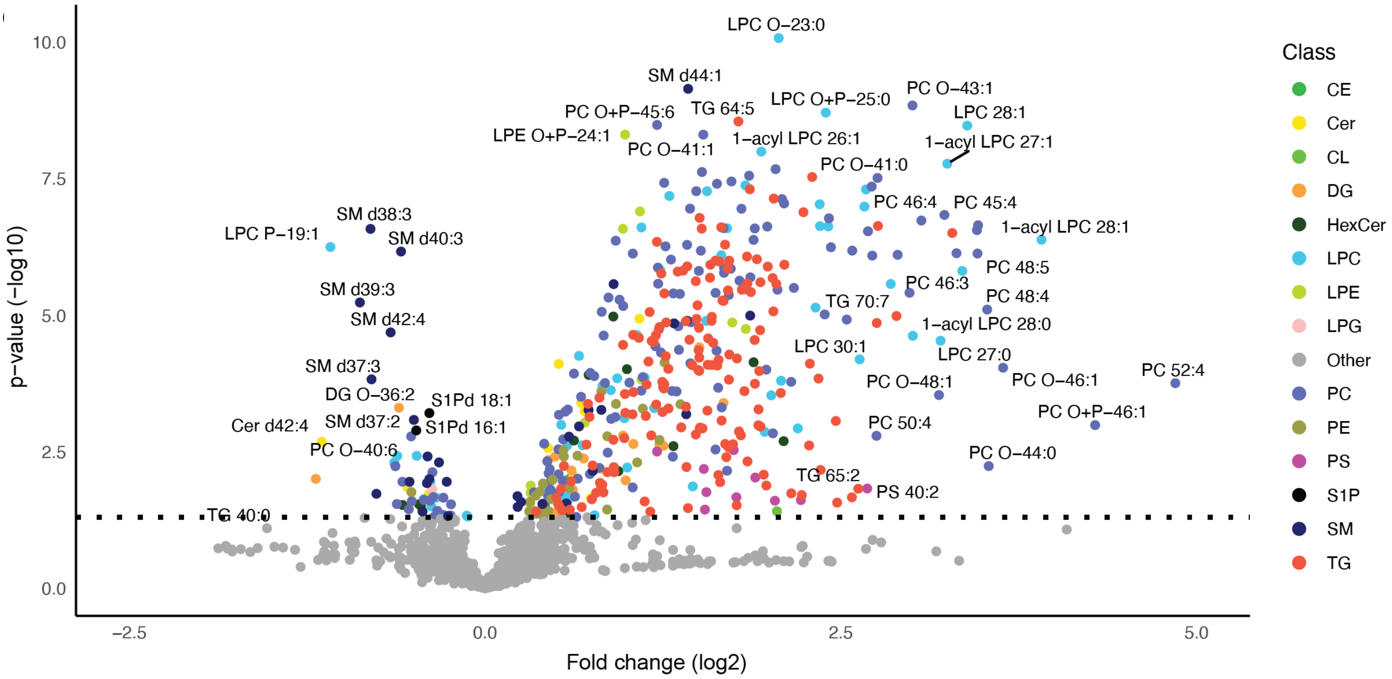

Legend: Volcano plot of lipid levels in male ALD patients with mild spinal cord disease (EDSS≤6) and aged >55 years versus controls aged >55 years. The vertical axis contains the p-value (−log10), and the horizontal axis the fold change (log2). Colored dots are lipids with a p-value of <0.05.

Supplemental Figure 2

Figure 2A: Subgroup analysis for male ALD patients based on the presence or absence of cerebral ALD and/or adrenal insufficiency (AI).

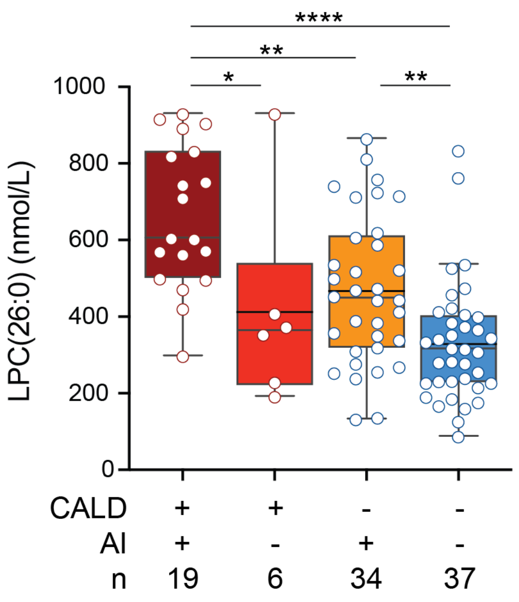

Legend: Targeted LPC(26:0) analysis in plasma of male ALD patients (n=96), CALD patients who underwent HCT were excluded from the analysis. Patients were grouped according to the presence or absence of cerebral ALD and/or adrenal insufficiency (AI) and number (n) of patients per group are indicated. The complete dataset is available in Supplementary Data 5. Kruskal-Wallis test was used to determine significant differences between groups, followed by Dunn's post-hoc test with FDR correction (*P≤0.05; **P≤0.01; ****P≤0.0001).

Figure 2B: Subgroup analysis for male ALD patients with mild or severe spinal cord disease grouped on the presence or absence of cerebral ALD and/or adrenal insufficiency (AI).

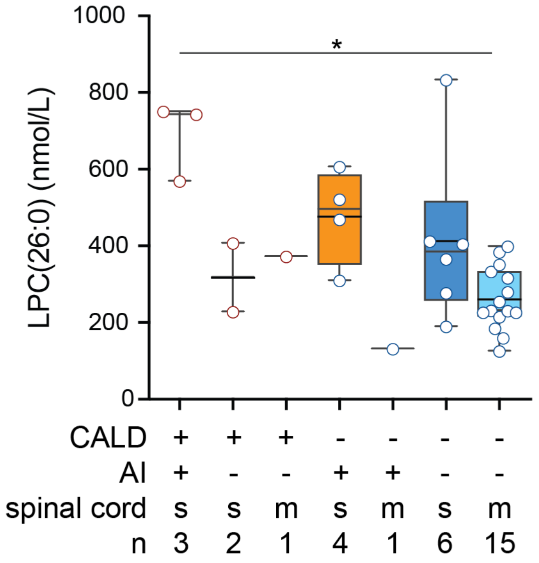

Legend: Targeted LPC(26:0) analysis in plasma of male ALD patients with mild or severe spinal cord disease (n=32). Patients were grouped according to the presence or absence of cerebral ALD, adrenal insufficiency (AI). Spinal cord involvement is mild (m, EDSS ≤6) or severe (s, EDSS >6). The number (n) of patients per group are indicated. The complete dataset is available in Supplementary Data 5. Kruskal-Wallis test was used to determine significant differences between groups, followed by Dunn's post-hoc test with FDR correction (*P≤0.05).
