# Supplementary material for: Lipidomic biomarkers in plasma correlate with disease severity in adrenoleukodystrophy
Source: Commun Med (Lond). 2024 Sep 10;4:175. doi: 10.1038/s43856-024-00605-9 (PMC11387402; doi:10.1038/s43856-024-00605-9)
Supplement: Supplementary file 3 — Description of Additional Supplementary Files [file 43856_2024_605_MOESM3_ESM.pdf]

Description of Additional Supplementary Files

File name: Supplementary Data 1

File description: Lipidomics data males.

File name: Supplementary Data 2

File description: Statistical results lipidomics data males.

File name: Supplementary Data 3

File description: Lipidomics data females.

File name: Supplementary Data 4

File description: Statistical results lipidomics data females.

File name: Supplementary Data 5

File description: Targeted LPC(26:0) data males and females.

File name: Supplementary Data 6

File description: Statistical results targeted LPC(26:0) data males and females.

File name: Supplementary Data 7

File description: VLCFA data males and females.

File name: Supplementary Data 8

File description: Statistical results VLCFA data males and females.
