# Supplementary material for: Lipidomic biomarkers in plasma correlate with disease severity in adrenoleukodystrophy
Source: Commun Med (Lond). 2024 Sep 10;4:175. doi: 10.1038/s43856-024-00605-9 (PMC11387402; doi:10.1038/s43856-024-00605-9)
Supplement: Supplementary file 12 — Reporting Summary [file 43856_2024_605_MOESM12_ESM.pdf]

nature portfolio

Corresponding author(s): Kemp

Last updated by author(s): Nov 29, 2023

Reporting Summary

Nature Portfolio wishes to improve the reproducibility of the work that we publish. This form provides structure for consistency and transparency in reporting. For further information on Nature Portfolio policies, see our Editorial Policies and the Editorial Policy Checklist.

Statistics

For all statistical analyses, confirm that the following items are present in the figure legend, table legend, main text, or Methods section.

n/a	Confirmed	
☐	☒	The exact sample size (*n*) for each experimental group/condition, given as a discrete number and unit of measurement
☐	☒	A statement on whether measurements were taken from distinct samples or whether the same sample was measured repeatedly
☐	☒	The statistical test(s) used AND whether they are one- or two-sided<br>*Only common tests should be described solely by name; describe more complex techniques in the Methods section.*
☐	☒	A description of all covariates tested
☐	☒	A description of any assumptions or corrections, such as tests of normality and adjustment for multiple comparisons
☐	☒	A full description of the statistical parameters including central tendency (e.g. means) or other basic estimates (e.g. regression coefficient) AND variation (e.g. standard deviation) or associated estimates of uncertainty (e.g. confidence intervals)
☒	☐	For null hypothesis testing, the test statistic (e.g. *F*, *t*, *r*) with confidence intervals, effect sizes, degrees of freedom and *P* value noted<br>*Give P values as exact values whenever suitable.*
☒	☐	For Bayesian analysis, information on the choice of priors and Markov chain Monte Carlo settings
☒	☐	For hierarchical and complex designs, identification of the appropriate level for tests and full reporting of outcomes
☒	☐	Estimates of effect sizes (e.g. Cohen's *d*, Pearson's *r*), indicating how they were calculated

Our web collection on statistics for biologists contains articles on many of the points above.

Software and code

Policy information about availability of computer code

Data collection	Thermo Scientific Xcalibur 4.3<br>Masslynx 4.2
Data analysis	lipidr 2.15.1<br>XCMS 3.14.1<br>Thermo Scientific Xcalibur 4.3<br>Graph Pad Prism version 9.5.1<br>R 4.3.1<br>R studio 2023.09.1<br>GGPLOT 3.4.4

For manuscripts utilizing custom algorithms or software that are central to the research but not yet described in published literature, software must be made available to editors and reviewers. We strongly encourage code deposition in a community repository (e.g. GitHub). See the Nature Portfolio guidelines for submitting code & software for further information.

Data

Policy information about availability of data

All manuscripts must include a data availability statement. This statement should provide the following information, where applicable:

- Accession codes, unique identifiers, or web links for publicly available datasets
- A description of any restrictions on data availability
- For clinical datasets or third party data, please ensure that the statement adheres to our policy

Data that support the findings of this study are available from the authors upon reasonable request

Human research participants

Policy information about studies involving human research participants and Sex and Gender in Research.

Reporting on sex and gender	The data on sex was available from clinical records and is reported in the manuscript. Data collection and analysis were done for males and females separately.
Population characteristics	Population characteristics that were used in the analysis include sex, age, presence of leukodsytrophy (cerebral ALD), presence of spinal cord disease (scored by EDSS) and the presence of adrenal insufficiency (defiined as patients who were receiving hormonal replacement therapy)
Recruitment	Plasma samples were collected from 24 healthy controls (12 male and 12 female) and 216 ALD patients (148 male and 68 female) from the biobank linked to the "Dutch ALD cohort", the German center of excellence for ALD in Leipzig and the university of Minnesota Division of Pediatric Blood and Marrow Transplant and Cellular Therapies. The selection of patients for the study was contingent upon the availability of suitable sample material and the procurement of informed consent for participation in scientific research.
Ethics oversight	Written informed consent was received from each patient. The study protocol was approved by the local Institutional Review Board (METC 2018–310).

Note that full information on the approval of the study protocol must also be provided in the manuscript.

Field-specific reporting

Please select the one below that is the best fit for your research. If you are not sure, read the appropriate sections before making your selection.

☒ Life sciences ☐ Behavioural & social sciences ☐ Ecological, evolutionary & environmental sciences

For a reference copy of the document with all sections, see nature.com/documents/nr-reporting-summary-flat.pdf

Life sciences study design

All studies must disclose on these points even when the disclosure is negative.

Sample size	Plasma samples were collected from 24 healthy controls (12 male and 12 female) and 216 ALD patients (148 male and 68 female). Sample sizes of specific groups that are included in the analysis are provided in the methods section in table 1.
Data exclusions	No data was excluded in this study.
Replication	Lipidomics analysis, targeted LPC(26:0) analysis and VLCFA analysis employed the use of standardized sample handling and the inclusion of quality control samples, along with system suitability tests to maintain data consistency and reliability. Targeted LPC(26:0) analysis was performed under the EN ISO 15189:2012 norm
Randomization	Data collection on all used analyses was done using a randomized batch design.
Blinding	Investigators were blinded to group allocation during data collection for the lipidomics analysis, targeted LPC(26:0) analysis and C26:0 analysis.

Reporting for specific materials, systems and methods

We require information from authors about some types of materials, experimental systems and methods used in many studies. Here, indicate whether each material, system or method listed is relevant to your study. If you are not sure if a list item applies to your research, read the appropriate section before selecting a response.

Materials & experimental systems

n/a	Involved in the study
☒	☐ Antibodies
☒	☐ Eukaryotic cell lines
☒	☐ Palaeontology and archaeology
☒	☐ Animals and other organisms
☐	☒ Clinical data
☒	☐ Dual use research of concern

Methods

n/a	Involved in the study
☒	☐ ChIP-seq
☒	☐ Flow cytometry
☒	☐ MRI-based neuroimaging

Clinical data

Policy information about clinical studies
All manuscripts should comply with the ICMJE guidelines for publication of clinical research and a completed CONSORT checklist must be included with all submissions.

Clinical trial registration	NA, this study was not a clinical trial
Study protocol	NA, this study was not a clinical trial
Data collection	Samples were obtained from the peroxisome biobank that is part of an ongoing prospective natural history study (METC 2018–310)
Outcomes	NA, this study was not a clinical trial
